# Effects of different volumes of ovariectomy on fertility and offspring development of rats

**DOI:** 10.3389/fendo.2023.1279610

**Published:** 2023-11-09

**Authors:** Yu Yang, Xiangyan Ruan, Jiaojiao Cheng, Xin Xu, Alfred O. Mueck

**Affiliations:** ^1^ Department of Gynecological Endocrinology, Beijing Obstetrics and Gynecology Hospital, Capital Medical University, Beijing Maternal and Child Health Care Hospital, Beijing, China; ^2^ Department for Women’s Health, University Women’s Hospital and Research Center for Women’s Health, University of Tuebingen, Tuebingen, Germany

**Keywords:** ovariectomy, fertility, premature ovarian insufficient, offspring, ovarian function

## Abstract

**Objective:**

The purpose of this study was to explore the effect of removal of different volumes of ovarian tissue on fertility and offspring development of SD rats.

**Methods:**

SD rats were randomly divided into 6 groups according to different volumes of ovariectomy: Sham group (n=6), non-ovariectomized; 25%-OVX group (n=6), with half of the left ovary excised; 50%-OVX group (n=5), with the left ovary excised; 75%-OVX group (n=5), with the left ovary and half of the right ovary excised; 87.5%-OVX group (n=6), with the left ovary and three quarters of the right ovary excised; 100%-OVX group (n=6), with bilateral ovaries excised. These female rats (F0) were mated with healthy male rats one and four months after the surgery, and the offspring of F0 rats were named F1^1mon^ and F1^4mon^, respectively. The number of days from mating to delivery and number of live cubs were recorded. At postnatal day 21 (P21), the body weight, length and anogenital distance (AGD) of the cubs were measured.

**Results:**

There were no differences in the number of live cubs between 25%-OVX, 50%-OVX and sham groups. Rats in the 87.5%-OVX group did not give birth at 1 month and 4 months after the operation. When compared with the sham group, the body weight and length of F1^1mon^ at P21 were increased in 25%-OVX group and 50%-OVX group. However, after the second delivery, we controlled each mother’s lactation to no more than eight pups. As a result, there were no differences in the body weight, length and AGD of F1^4mon^ compared with sham group.

**Conclusion:**

Removal of less than 50% of the ovaries did not affect the fertility of rats and offspring development of rats.

## 1 Introduction

Premature ovarian insufficient (POI) refers to the loss of ovarian activity before the age of 40 (1). POI has adverse effects on women’s fertility, psychological and sexual health, as well as bone, cardiovascular and cognitive health (2–4). Conditions such as endometriosis, ovarian cyst, teratoma, corpus luteum rupture, etc. often require excision of the lesion and may even necessitate ovariectomy, resulting in diminished ovarian reserve and even iatrogenic POI (5). The most ideal situation is one in which sufficient ovarian tissue can be preserved while also completely removing the lesions. Therefore, we aim to determine the extent of ovarian tissue that can be removed without damaging the women’s endocrine function and fertility.

In addition to gonadal surgery, POI can also be caused by gonadotoxic therapies such as radiotherapy and chemotherapy. It is no longer an experimental technique to preserve ovarian function by cryopreservation of ovarian tissue before gonadal toxicity treatment (6). While some patients may not need gonadotoxic treatment at present, but the condition is changing, in the process of disease development, it may be necessary to initiate gonadotoxic treatment at any time. For these patients, having a portion of ovarian tissue frozen provides the advantage of maintaining normal endocrine and reproductive functions with the remaining ovaries in the body when the disease is stable. Once it is determined that gonadotoxic treatment needs to be initiated, it can be started immediately without delaying treatment due to fertility protection. Therefore, our aim is to collect and preserve as much ovarian tissue as possible through cryopreservation, ensuring an adequate fertility potential for patients. At the same time, we also hope that the ovaries remained in the body can function normally before the initiation of gonadotoxic treatment.

Previous research by our team discovered that the removal of up to 75% of ovarian tissue did not impact the secretion of estradiol and progesterone in rats (7). The purpose of this study is to further explore the maximum volume of ovarian tissue that can be removed without adversely affecting the fertility of rats.

## 2 Study design

### 2.1 Feeding of animals

Healthy female specific-pathogen-free grade Sprague-Dawley (SD) rats, aged 7 to 8 weeks, were obtained from Beijing Vital River Laboratory Animal Technology Company Limited (Beijing, China). The animal license is SCXK (Beijing, China) 2016-0011. In the Laboratory Animal Centre at Capital Medical University, China, 2 to 3 rats were housed in each cage. The animals had free access to both water and food. All rats were maintained under controlled conditions, including 12-h light/dark cycle, a temperature ranging from 19 ~ 24 °C and a relative humidity of 40 ~ 70%. The study was approved from the Animal Experiments and Experimental Animal Welfare Committee of Capital Medical University, China (AEEI-2021-146).

### 2.2 Grouping of experimental animals

**Figure 1:**
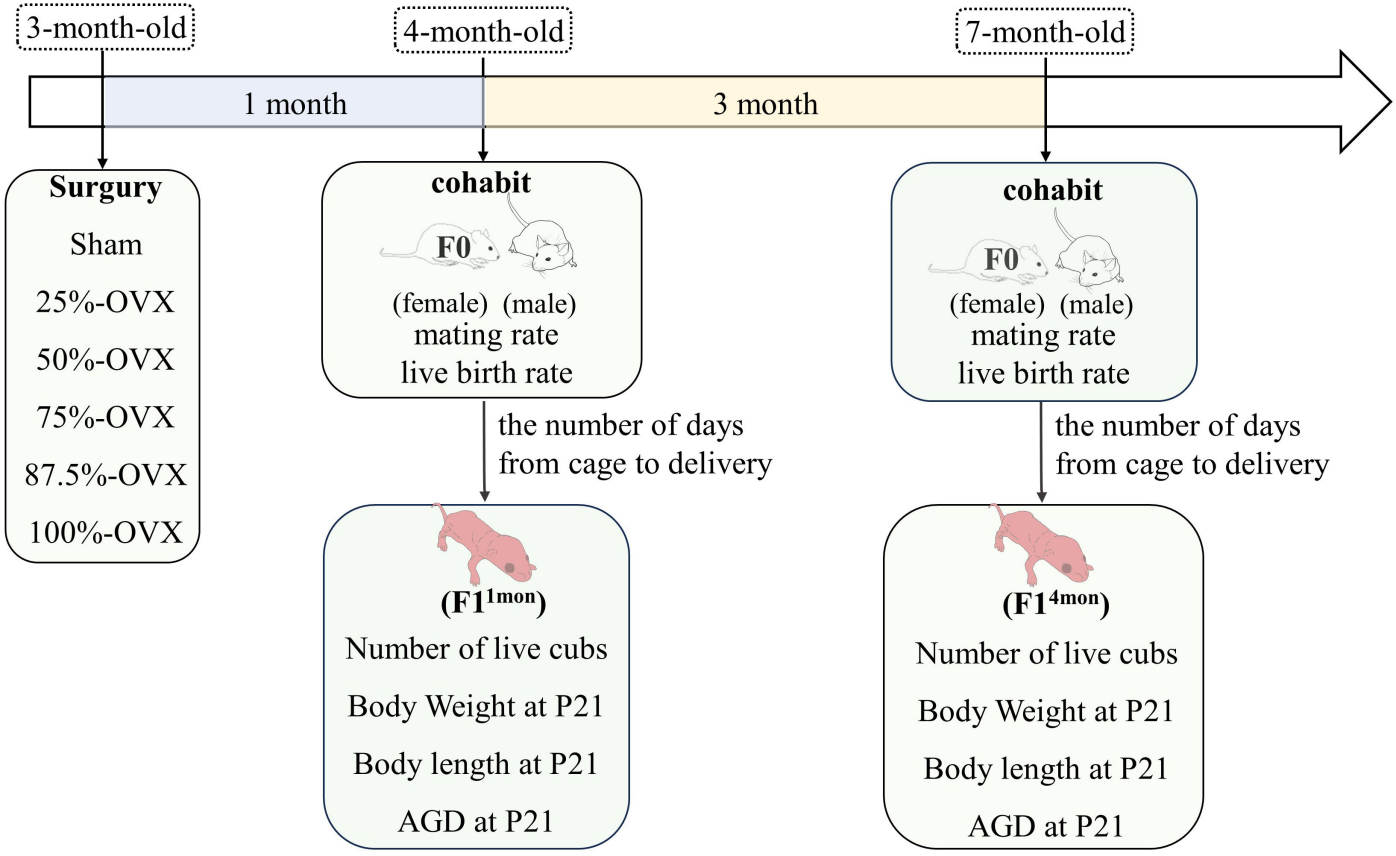
Flow chart of the study. AGD, anogenital distance. P21, 21 days postpartum.

Vaginal smears were performed daily from 8 am to 10 am for a period of 10 days following a 10-day acclimatization period. Rats with a normal estrous cycle were included in the study. In total, 36 rats (F0) were randomly divided into six groups according to the volume of ovariectomy (OVX): ① Sham group (n=6). ② 25%-OVX group (n=6), with half of the left ovary excised. ③ 50%-OVX group, with the left ovary excised (an animal died of anesthesia, and leaving five rats for subsequent experiments). ④ 75%-OVX group, with the left ovary and half of the right ovary excised (an animal died of anesthesia, and leaving five rats for subsequent experiments). ⑤ 87.5%-OVX group, with the left ovary and three quarters of the right ovary excised (n=6). ⑥ 100%-OVX group, with bilateral ovariectomy (n=6). The sham operation group was the control group, and the other 5 groups were experimental group. The specific surgical procedure was identical to that described in our previous study (7). The surgery was performed at when the F0 rats reached 3 months of age. The body weight of F0 rats was weighed weekly until 1 month after the surgery. The experimental flow chart is shown in **Figure 1**.

### 2.3 Fertility and offspring development evaluation

One month after the surgery, female F0 rats from the six groups were caged with healthy male rats. Two female F0 rats were placed in the same cage as one healthy male rat(cohabitation). After that, vaginal smears were performed each morning from 8:00 to 10:00 am. Finding vaginal plugs or sperm was considered to be successful mating. If no mating occurred for 10 consecutive days, the female rat was placed into the cage of another male rat. If the female rat remained unmated for another 10 days following the cage change, it is considered to be incapable of mating. Once the female rats were found mating, they were isolated in a separate cage until delivery. The interval between cohabitation and delivery as well as the number of live cubs (F1), were recorded for each F0 female. This process was repeated four months after the surgery. The offspring of F0 female rats that mated one month and four months after surgery were named F1^1mon^ and F1^4mon^, respectively. The body length, body weight and anogenital distance (AGD) of F1 rats were measured at 21 days postpartum (P21). The experimental flow chart is shown in **Figure 1**.

### 2.4 Statistical analysis

The results are presented as mean ± s.e.m. IBM SPSS Statistics 26.0 was used for statistical analysis and GraphPad Prism 8.0. 2 was used for creating graphs. The Shapiro-Wilk test was used to assess whether the data followed a normal distribution. For data that exhibited both with normal distribution and homogeneous variance, one-way ANOVA was used for analysis, followed by the Dunnett test for post-comparison. Kruskal- Wallis test was used for non-normal distribution or heterogeneity of variance. A significance level of *P* < 0.05 was considered statistically significant.

## 3 Results

### 3.1 The changes of body weight in F0 rats after operation

**Figure 2:**
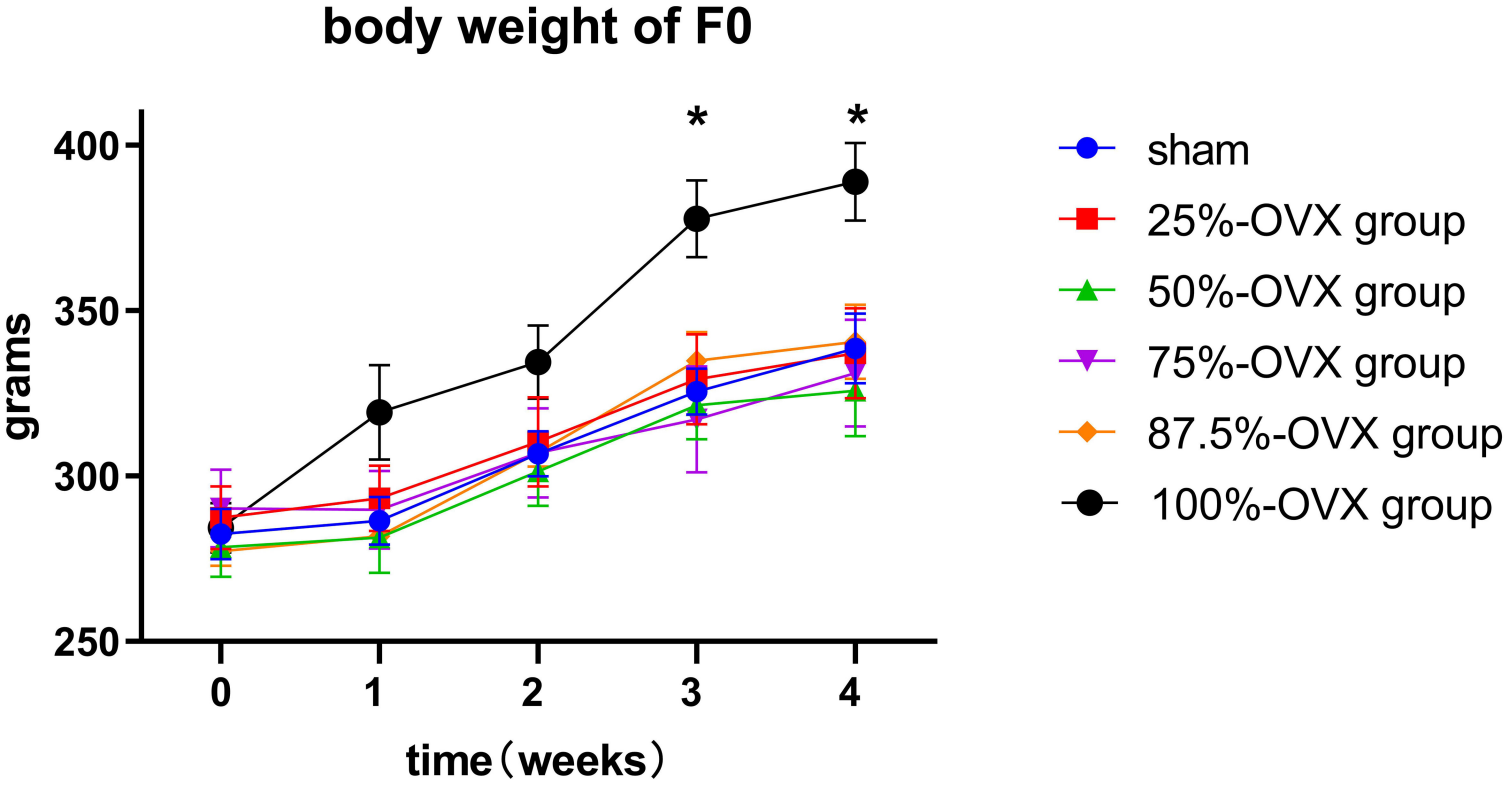
Body weight of F0 rats before surgery, 1 week, 2 weeks, 3 weeks and 4 weeks after surgery. *, p < 0.05.

There were no difference in the body weight of F0 rats among the 6 groups from baseline (before surgery) to 2 weeks postoperatively. However, at 3 weeks (377.78 ± 11.56 g) and 4 weeks (388.98 ± 11.71 g) postoperatively, the 100%-OVX group exhibited higher body weight compared to the sham group (3 weeks postoperatively 325.57 ± 6.91 g, 4 weeks postoperatively 338.55 ± 10.59 g) (*P*=0. 014 at 3 weeks postoperatively, *P*=0. 041 at 4 weeks postoperatively). The body weight in the 25%-OVX, 50%-OVX, 75%-OVX and 87.5%-OVX groups did not show statistically significant differences compared to the sham group. The body weight of F0 rats in each group is shown in **Figure 2**.

### 3.2 Mating rate and live birth rate of F0 rats after operation

In 100%-OVX group, none of the F0 female rats successfully mated, while no significant difference in mating rates was observed among the other five groups. Within the 87.5%-OVX group of F0 female rats, there were no instances of live births at 1 month and 4 months after the surgery. Only one female rat in the 75%-OVX group had live birth 1 month after the surgery, but there was no live birth in this group four months after operation. There were no statistically significant differences in live birth rates among the Sham Group, the 25%-OVX Group and the 50%-OVX Group at 1 month and 4 months after the surgery. The mating rate and live birth rate of F0 rats at 1 month and 4 months after the surgery are shown in **Table 1**.

**Table 1:** Mating rate and live birth rate of F0 rats 1 month and 4 months after operation.

	1 month after operation			4 months after operation		
	Mating rate (n1/N)	Live birth rate (n2/N)	Number of live cubs	Mating rate (n1/N)	Live birth rate (n2/N)	Number of live cubs
sham	100%(6/6)	83.33%(5/6)	14.60 ± 1.57	80%(4/5)	60%(3/5)	6.67 ± 3.18
25%-OVX	100%(6/6)	83.33%(5/6)	8.20 ± 2.18	60%(3/5)	40%(2/5)	8.50 ± 4.50
50%-OVX	100%(5/5)	60%(3/5)	12.00 ± 0.58	80%(4/5)	60%(3/5)	8.00 ± 1.16
75%-OVX	60% (3/5)	20%(1/5)	11	100%(4/4)	0%(0/4)	\
87.5%-OVX	66.7%(4/6)	0%(0/6)	\	16.67%(1/6)	0%(0/6)	\
100%-OVX	0% (0/6)	0%(0/6)	\	0%(0/6)	0%(0/6)	\

n1 means the number of F0 females that mated successfully, n2 means the number of F0 females that had live births, and N means the sample size of each group.

### 3.3 Time from cohabitation to delivery

**Figure 3:**
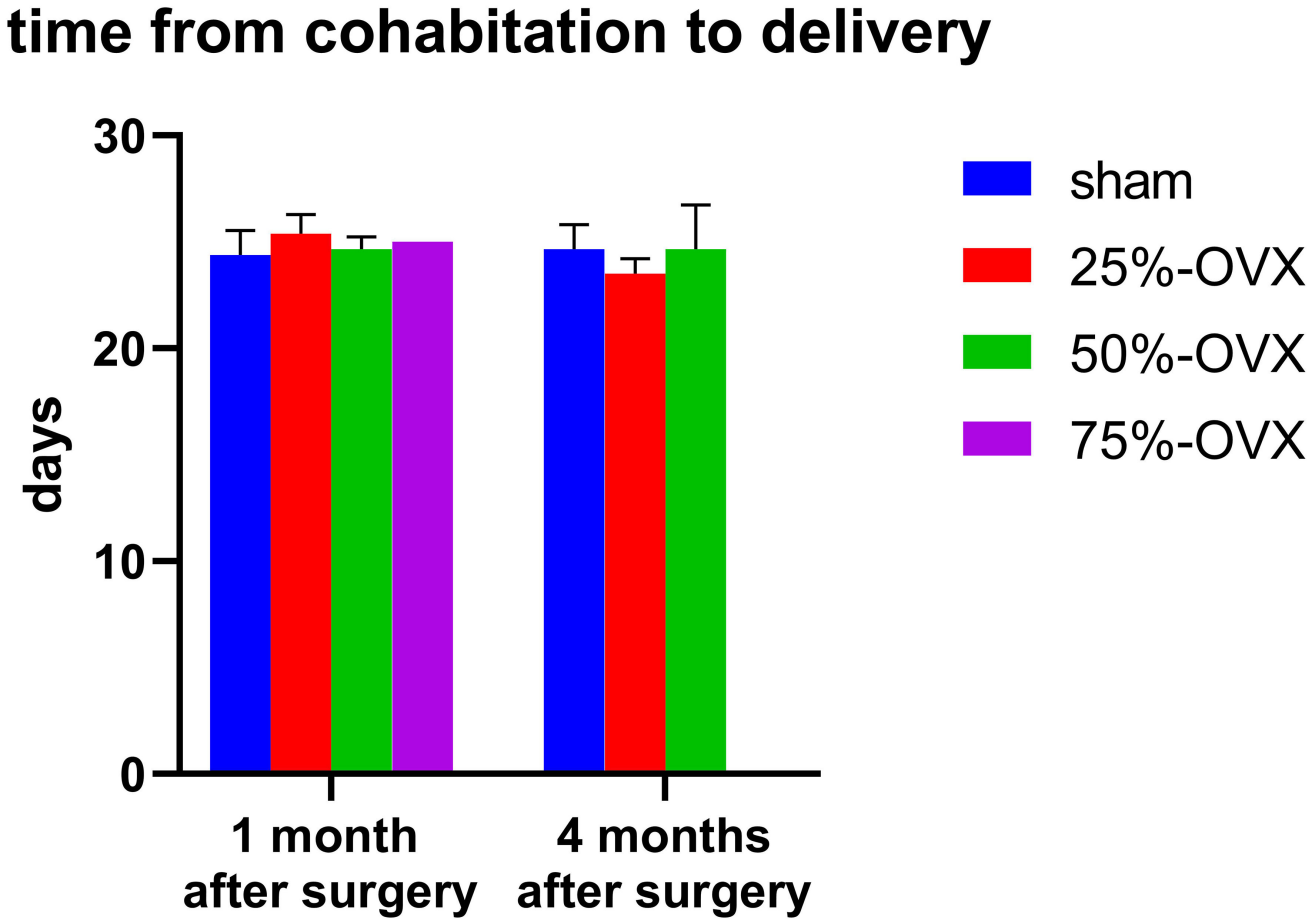
Days from cohabitation to delivery of F0 female rats 1 and 4 months postoperatively.

One month after the surgery, the time from cohabitation to delivery in the sham, the 25%-OVX, the 50%-OVX groups was 24.40 ± 0.51, 25.40 ± 0.40, 24.67 ± 0.33 days, respectively. There were no significant differences among the three groups (P=0. 265). In the 75%-OVX group, only one female rat had a live birth, taking 25 days from cohabitation to live birth. Four months after operation, the time from cohabitation to delivery in the sham, the 25%-OVX, the 50%-OVX groups was 24.67 ± 0.67, 23.50 ± 0.50, 24.67 ± 1.20, respectively. Again, there were no significant differences among these three groups (P=0. 528). (**Figure 3**).

### 3.4 Litter size of F0 rats

One month after the surgery, the number of live litters in the 25%-OVX group (8.20 ± 2.18) and the 50%-OVX group(12.00 ± 0.58) did not show statistically significant differences compared to the sham group(14.60 ± 1.57) (P=0. 166). In the 75%-OVX group, the only female rat with live birth delivered 11 cubs. Four months after the surgery, the number of live litters in the sham group, the 25%-OVX group and the 50%-OVX group was 6.67 ± 3.18, 8.50 ± 4.50 and 8.00 ± 1.16, respectively, with no differences among the three groups (p=0. 900). The data are shown in **Table 1**.

### 3.5 Body weight, body length, AGD of F1 rats at P21

The body weight, and body length of F1^1mon^ in 25%-OVX(74.92 ± 1.15g, 13.95 ± 0.07cm) and 50%-OVX group (65.50 ± 1.75g, 13.87 ± 0.08cm) were higher than those of the sham group (59.20 ± 0.96g, 13.05 ± 0.10cm) at P21. AGD of male F1^1mon^ in 25%-OVX group(21.30 ± 0.31mm) was higher the sham group(19.56 ± 0.25mm) at P21. But there was no difference in AGD of female F1^1mon^ among the three groups(p=0. 123). Considering that different litter sizes may impact the development of cubs during the lactation period, each female rat retained no more than 8 cubs for lactation and the surplus litters were sacrificed on the day of second delivery. After the above procedure, the differences in the body weight, length and AGD disappeared in F1^4mon^. The Data are shown in **Table 2**.

**Table 2:** The body weight, length and AGD of F1 offspring after 21 days of lactation.

	F1^1mon^				F1^4mon^			
	Weight (g)	Length(cm)	Female AGD (mm)	Male AGD (mm)	Weight (g)	Length (cm)	Female AGD (mm)	Male AGD (mm)
Sham	59.20 ± 0.96	13.05 ± 0.10	12.51 ± 0.16	19.56 ± 0.25	80.15 ± 2.96	14.08 ± 0.19	14.00 ± 0.32	22.14 ± 0.55
25%-OVX	74.92 ± 1.15***	13.95 ± 0.07***	13.18 ± 0.31	21.30 ± 0.31***	82.74 ± 2.30	13.98 ± 0.13	14.69 ± 0.21	23.13 ± 0.72
50%-OVX	65.50 ± 1.75**	13.87 ± 0.08***	12.59 ± 0.27	20.69 ± 0.40	87.42 ± 1.07	14.54 ± 0.08	14.17 ± 0.48	22.00 ± 0.27

F1^1mon^, the offsprings which delivered by F0 female rats 1 month postoperatively. F1^4mon^, the offsprings which delivered by F0 female rats 4 month postoperatively. AGD, anogenital distance. **, compared with sham group, P< 0.01; ***, compared with sham group P< 0.001.

## 4 Discussion

### 4.1 Effect of partial ovariectomy on fertility in rats

Our previous studies discovered that even up to 75% of the total ovarian tissue can be removed without impacting E2 and P production in rats, and 50% ovarian tissue resection did not affect the levels of anti-mullerian hormone, inhibin B and follicle stimulating hormone (7). In this study, we have shown that female rats lose their mating ability after bilateral ovariectomy, and even with the retention of only 12.5% of ovarian tissue, they are still able to mate. When the resection volume reached 75% of the total ovarian tissue, the rats could maintain fertility for up to 1 month postoperatively, but their fertility would be lost 4 months after the surgery. When no more than 50% of total ovarian tissue was removed, the mating behavior, gestation days and the number of live cubs in SD rats were not affected at both 1 and 4 months postoperatively. *Rahima et al.* found that the number of implantation sites and live fetuses in unilateral ovariectomy (ULO) group was not statistically different from the control group (with intact ovaries) on days 13, 16 and 22 of gestation in Wistar rats (8). Our study further observed live births and did not find any effect of unilateral ovariectomy on the number of live cubs.

Clinical studies have been conducted on the effect of unilateral oophorectomy on fertility in human women. The age of menopause in women with unilateral ovariectomy is about 1 to 2 years earlier than that in women with both ovaries intact (9, 10). The study of *Bellati et al.* showed that there was no statistical difference in the fertility outcomes of women after unilateral ovariectomy compared with those after appendectomy or cholecystectomy (11). However, the maximum amount of ovarian tissue that can be removed without affecting a woman’s fertility has not been studied.

### 4.2 Effect of partial ovariectomy on body weight and length of F1 offsprings

In *Rahima’s* study, fetal weights in ULO group were similar to the control group on Days 13,16 and 22 of gestation, implying that unilateral ovariectomy does not affect the development of embryos in the uterine cavity (12). In our study, although the weight and length of F1^1mon^ in the 25%-OVX and 50%-OVX groups were larger than those in the sham group, when the number of lactating pups were adjusted for in F1^4mon^, the differences were disappeared, suggesting that partial ovariectomy does not have adverse effects on fetal development.

### 4.3 Effect of partial ovariectomy on offspring’s AGD

AGD can be used as a sensitive index to evaluate the degree of masculinization and predict the abnormal development of reproductive system (13, 14). Male AGD is obviously longer than the female AGD, making AGD a marker for gender identification (15). Exposure to antiandrogens and estrogens during pregnancy can shorten AGD in offspring (14). Studies have shown that shortened AGD is related to cryptorchidism, hypospadias and decline in sperm quality during adulthood (16–18). However, androgen exposure during pregnancy will cause prolonged AGD in offspring, resulting in polycystic ovary syndrome-like phenotype in female offspring (19). The AGD of male F1^1mon^ in the 25%-OVX group was slightly higher than that in Sham group at P21, but there was no difference in the AGD of F1^4mon^ among the sham, 25%-OVX and 50%-OVX groups, for both female or male offspring. This suggestes that partial ovariectomy did not affect the level of androgen in pregnant rats, and did not cause adverse effects on the developmental processes of the offspring’s reproductive system.

### 4.4 Effect of partial ovariectomy on body weight of F0 rats

The ovary is not only related to normal reproductive endocrine function, but it is also very important for metabolic health (20–22). The body weight of rats after bilateral ovariectomy was significantly higher than that of the sham operation group 3 weeks later, which showed that the metabolic effect of bilateral ovariectomy could occur quickly after sudden drop in estrogen levels. However, the removal of 87.5% of ovarian tissue did not affect the weight of rats, so the attention should be paid to the protection of normal ovary during the surgery. Even if only 12.5% of normal ovarian tissue can be retained, it will help to maintain women’s metabolic health and reduce the occurrence of obesity.

### 4.5 Compensatory changes of residual ovary after partial ovariectomy

After unilateral ovariectomy, compensatory hypertrophy of the contralateral ovary occurs, and the number of ovulations of the remaining contralateral ovary increase to a similar level to that of animals with intact bilateral ovaries. This phenomenon has been reported in mice, hamsters, rabbits, cats and pigs (23). However, when we increased the volume of the removed ovary to 75%, only one rat had live birth one month after the surgery, and no rats had live birth four months after the surgery. When the volume was further increased to 87.5%, no rats could achieve live birth one month after the surgery, indicating that retaining only 25% of the ovary is not enough to achieve compensation.

*Kagabu et al.* discovered that one week after unilateral ovariectomy, the number of large follicles in the contralateral ovary was similar to the sum of the number of large follicles in bilateral ovaries of sham-operated Wistar rats (23). But the count of small and medium-sized follicles (diameter 250~549um) was lower than that in the control group from 1 to 4 weeks postoperatively. 5 weeks later, the number of small and medium-sized follicles in the unilateral ovariectomy group was similar to that in the control group. These indicates that after unilateral ovariectomy, the compensatory increase in large follicles in the contralateral ovary precedes that of small follicles. *Kagabu’s research* also found that in unilateral ovariectomized rats, the proportion of early atresia follicles with diameters of 400-499μm (31.1%) was significantly lower compared to control rats (73.9%). This suggests that after unilateral ovariectomy, follicles immediately evade the fate of atresia and transverse the critical point of follicular development to become large ovulatory follicles.

### 4.6 Effects of maternal sex hormones on offspring health

To date, there have been no studies on the effects of removal of different volumes of ovarian tissue on the health of rat offspring. However, maternal hormone levels can affect the health of offspring. In recent years, studies have found that offspring born through assisted reproductive technology may have an increased risk of thyroid dysfunction, dyslipidemia and diminished verbal ability with exposure to supraphysiological estradiol *in utero* (24–26). Ovarian hyperstimulation syndrome(OHSS) offspring displayed reduced intellectual ability and more cardiovascular dysfunctions compared with non-OHSS offspring (27, 28). Intrauterine androgen exposure can also have adverse effects on offspring, such as lipid disorder, increased fasting glucose and impaired glucose tolerance, polycystic phenotype, lower muscle strength, delayed puberty and impaired ovarian reserve (29–33). Polycystic phenotypes due to androgen exposure can even be observed in F3 offspring (19). So, further research is needed on whether gonadal surgery can affect the health of future generation by altered maternal estrogen, androgens during pregnancy.

There is no standard for the amount of ovarian biopsy for ovarian tissue cryopreservation. Usually at least 1/2 of a unilateral ovary is taken. The results of this study suggest that when the amount of ovarian tissue removal does not exceed half of the total ovarian volume, the fertility of rats remain unaffected. If more clinical studies in the future confirm that this conclusion is also applicable to human women, half of the total ovarian tissue can be removed for ovarian biopsy, which can preserve fertility potential for patients as much as possible, without affecting the normal function of the ovary remaining in the body.

## 5 Limitation

Because there are many groups, the sample size of each group is limited. In 75%-OVX group, only one female rat has live birth one month after operation, which could not be compared with other groups. Although our study found that unilateral ovariectomy did not affect fertility in SD rats, previous studies have also found that the removal of 75% of the ovarian tissue did not affect the production of estrogen in rats, and unilateral ovariectomy did not affect the levels of anti-mullerian hormone, inhibin B and follicle stimulating hormone. It is important to note that this only applies to surgical premature ovarian insufficiency. The minimum amount of estrogen and other hormones to prevent the onset of menopause is still unknown.

## 6 Conclusion

When the amount of ovarian tissue removal does not exceed half of the total ovarian volume, the fertility and offspring development of rats remain unaffected. Therefore, in gonadal surgery, it is advisable to retain one side of the ovary as extensively as possible to preserve the normal fertility of women.

## Data availability statement

The raw data supporting the conclusions of this article will be made available by the authors, without undue reservation.

## Ethics statement

The animal study was approved by Ethics Committee on Animal Research of the Capital Medical University (ethics NO: AEEI-2021-146). The study was conducted in accordance with the local legislation and institutional requirements.

## Author contributions

YY: Writing – review & editing, Data curation, Formal Analysis, Investigation, Software, Writing – original draft. XR: Writing – review & editing. JC: Investigation, Writing – review & editing. XX: Investigation, Writing – review & editing. AM: Writing – review & editing.
